# What causes symptoms suggestive of tuberculosis in HIV-positive people with negative initial investigations?

**DOI:** 10.5588/ijtld.18.0251

**Published:** 2019-01-24

**Authors:** Y. Hanifa, S. Toro Silva, A. Karstaedt, F. Sahid, S. Charalambous, V. N. Chihota, G. J. Churchyard, A. von Gottberg, K. McCarthy, M. P. Nicol, N. T. Ndlovu, W. Stevens, K. L. Fielding, A. D. Grant

**Affiliations:** * TB Centre, London School of Hygiene & Tropical Medicine, London, UK; † Department of Medicine, Chris Hani Baragwanath Hospital, Johannesburg; ‡ University of the Witwatersrand, Johannesburg; § The Aurum Institute, Johannesburg; ¶ School of Public Health, Faculty of Health Sciences, University of the Witwatersrand, Johannesburg; # Advancing Care and Treatment for TB-HIV, South African Medical Research Council Collaborating Centre for HIV and TB, Tygerberg; ** Centre for Respiratory Diseases and Meningitis, National Institute for Communicable Diseases, Johannesburg; †† School of Pathology, Faculty of Health Sciences, University of the Witwatersrand, Johannesburg; ‡‡ Division of Medical Microbiology, Faculty of Health Sciences, University of Cape Town, Cape Town; §§ National Health Laboratory Service, Johannesburg; ¶¶ Department of Molecular Medicine and Haematology, School of Pathology, Faculty of Health Sciences, University of the Witwatersrand, Johannesburg; ## Africa Health Research Institute, School of Nursing and Public Health, University of KwaZulu-Natal, Durban, South Africa

**Keywords:** South Africa, Xpert^®^ MTB/RIF, TB symptoms, human immunodeficiency virus

## Abstract

**OBJECTIVE:**

To identify the causes of symptoms suggestive of tuberculosis (TB) among people living with the human immunodeficiency virus (PLHIV) in South Africa.

**METHODS:**

A consecutive sample of HIV clinic attendees with symptoms suggestive of TB (⩾1 of cough, weight loss, fever or night sweats) at enrolment and at 3 months, and negative initial TB investigations, were systematically evaluated with standard protocols and diagnoses assigned using standard criteria. TB was ‘confirmed’ if Mycobacterium tuberculosis was identified within 6 months of enrolment, and ‘clinical’ if treatment started without microbiological confirmation.

**RESULTS:**

Among 103 participants, 50/103 were preantiretroviral therapy (ART) and 53/103 were on ART; respectively 68% vs. 79% were female; the median age was 35 vs. 45 years; the median CD4 count was 311 vs. 508 cells/mm^3^. Seventy-two (70%) had ⩾5% measured weight loss and 50 (49%) had cough. The most common final diagnoses were weight loss due to severe food insecurity (*n* = 20, 19%), TB (*n* = 14, 14%: confirmed *n =* 7; clinical *n* = 7), other respiratory tract infection (*n* = 14, 14%) and post-TB lung disease (*n* = 9, 9%). The basis for TB diagnosis was imaging (*n* = 7), bacteriological confirmation from sputum (*n* = 4), histology, lumbar puncture and other (*n* = 1 each).

**CONCLUSION:**

PLHIV with persistent TB symptoms require further evaluation for TB using all available modalities, and for food insecurity in those with weight loss.

## Abstract (FR)

**OBJECTIF:**

Identifier les causes des symptômes suggestifs de tuberculose (TB) parmi les personnes vivant avec le virus de l'immunodéficience humaine (PVVIH) en Afrique du Sud.

**MÉTHODE:**

Unéchantillon consécutif de patients d'un dispensaire VIH ayant des symptômes suggestifs de TB (⩾1 parmi toux, perte de poids, fièvre ou sueurs nocturnes) lors de l'enrôlement età 3 mois, mais des premières investigationsà la recherche de TB négatives, aété systématiquementévalué grâceà des protocoles standard et des diagnostics assignés grâce aux critères standard. La TB aété « confirmée » lorsque Mycobacterium tuberculosis aété identifié dans les 6 mois de l'enrôlement, et « clinique » si le traitement a démarré sans confirmation microbiologique.

**RÉSULTATS:**

Parmi 103 participants, 50/103 pré ART et 53/103 sous ART, respectivement ; 68% contre 79% ontété des femmes ; d'âge médian 35 contre 45 ans, et le taux médian de CD4 de 311 contre 508 cellules/mm^3^. Soixante-douze patients (70%) ont eu une perte de poids mesurée ⩾5% et 50 (49%) une toux. Les diagnostics finaux les plus fréquents ontété une perte de poids dueà une insécurité alimentaire majeure (*n* = 20, 19%), une TB (*n* = 14, 14% : confirmée, *n* = 7 ; clinique, *n* = 7), d'autres infections des voies respiratoires (*n* = 14, 14%), une affection pulmonaire post-TB (*n* = 9, 9%). La base du diagnostic de TB aété l'imagerie (*n* = 7), la confirmation bactériologique des crachats (*n* = 4), l'histologie, la ponction lombaire et autres (*n* = 1 chacun).

**CONCLUSION:**

Les PVVIH ayant des symptômes de TB persistants requièrent davantage d'évaluationà la recherche de TB avec toutes les modalités disponibles et à la recherche de problèmes de sécurité alimentaire chez ceux qui ont perdu du poids.

## Abstract (ES)

**MARCO DE REFERENCIA:**

El estudio tuvo por objeto reconocer las causas de los síntomas indicativos de tuberculosis (TB) en las personas infectadas por el virus de la inmunodeficiencia humana (PLVIH) en Suráfrica.

**MÉTODO:**

Una muestra consecutiva de pacientes que acudían a la consulta del VIH y presentaban síntomas indicativos de TB (uno o varios síntomas como tos, pérdida de peso, fiebre o sudoración nocturna) en el momento de la inscripción y a los 3 meses y cuyas investigaciones iniciales de la TB habían sido negativas se evaluó de manera sistemática mediante protocolos normalizados y los diagnósticos se asignaron según criterios definidos. El diagnóstico de TB se consideró ‘confirmado’ cuando se detectaba el Mycobacterium tuberculosis en los primeros 6 meses después de la inscripción en la consulta y ‘clínico’ cuando se iniciaba tratamiento sin confirmación microbiológica.

**RESULTADOS:**

De los 103 participantes, 50 no recibían aun tratamiento antirretrovírico y 53 ya lo recibían; respectivamente, el 68% y el 79% eran de sexo femenino, la mediana de la edad era 35 y 45 años; la mediana de la cifra de linfocitos CD4 fue 311 y 508 células/mm^3^. Setenta y dos pacientes (70%) presentaron una pérdida de peso cuantificada ⩾5% y 50 presentaron tos (49%). Los diagnósticos definitivos más frecuentes fueron pérdida de peso debida a una inseguridad alimentaria grave en pacientes (*n* = 20, 19%), TB (*n* = 14, 14%: 7 diagnósticos confirmados y 7 clínicos), otras infecciones de las vías respiratorias (*n* = 14, 14%) y enfermedad pulmonar crónica posterior a la TB (*n* = 9, 9%). El diagnóstico de TB se fundamentó en las imágenes (*n* = 7), la confirmación bacteriológica en el esputo (*n* = 4), el examen histológico, la punción lumbar y otros medios (*n* = 1, en cada caso).

**CONCLUSIÓN:**

Es necesario investigar la presencia de TB en las PLVIH y síntomas persistentes indicativos de TB, mediante todos los medios al alcance y la inseguridad alimentaria en las personas que presentan pérdida de peso.

THE WORLD HEALTH ORGANIZATION (WHO) recommends regular screening of people living with the human immunodeficiency virus (PLHIV) for tuberculosis (TB) using a symptom screen comprising any one of current self-reported cough, fever, weight loss or night sweats (hereafter termed the ‘WHO tool’), as an essential part of the HIV care package.^1^ Although people attending for HIV care in sub-Saharan Africa are highly symptomatic,^2^ most of those reporting WHO tool symptoms have negative TB investigations,^2–4^ and a proportion continue to report symptoms. Early identification of people with active TB among PLHIV is a priority; however, the evidence underpinning investigation pathways after an initial sputum test is weak.^5–14^

The aim of our study was to determine the causes of persistent or recurrent symptoms suggestive of TB among ambulatory adults attending for HIV care who had negative initial TB investigations.

## METHODS

This sub-study was part of a prospective cohort study evaluating a risk-based algorithm to prioritise Xpert^®^ MTB/RIF (Cepheid, Sunnyvale, CA, USA) testing among adults attending for routine HIV care in South Africa: ‘Xpert for people attending HIV/AIDS care: test or review?’ (XPHACTOR).^15^

### XPHACTOR study population, recruitment and procedures

**Figure A.1:**
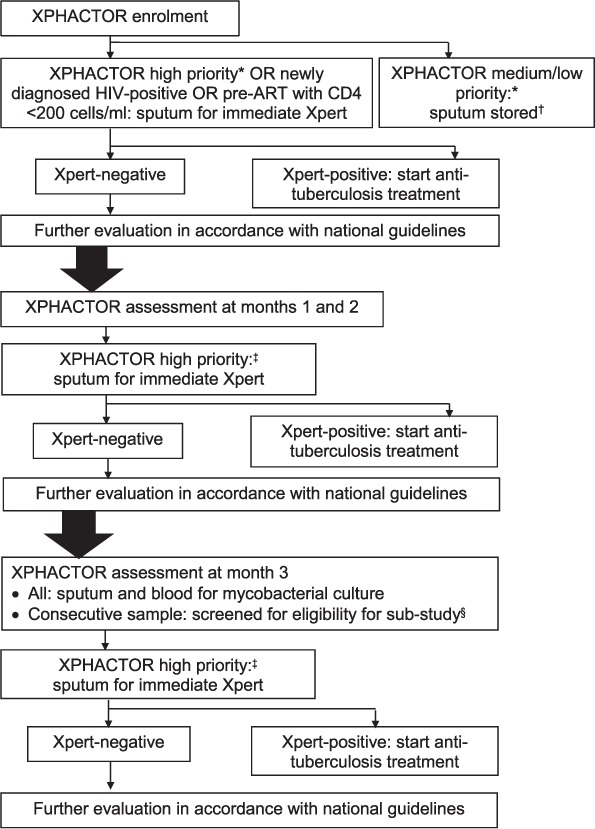
XPHACTOR main study flow and procedures. ^*^ XPHACTOR algorithm at enrolment: high priority (any of current cough, fever ⩾3 weeks, BMI < 18.5 kg/m^2^, CD4 < 100×10^6^/l, measured weight loss ⩾10% in preceding 6 months or other feature raising high clinical suspicion of TB); medium priority (any of fever <3 weeks, night sweats, measured weight loss <10% in preceding 6 months); low priority = no TB symptoms. ^†^ Samples tested using Xpert at the end of the study to enable comparison of sensitivity and specificity of the XPHACTOR study algorithm to detect TB cases against sensitivity and specificity if Xpert had been performed immediately for all with any WHO tool symptom. ^‡^ XPHACTOR algorithm at monthly follow-up: high priority (any of current cough, fever ⩾3 weeks, night sweats ⩾4 weeks, BMI < 18.5 kg/m^2^, CD4 < 100×10^6^/l, measured weight loss ⩾10% in preceding 6 months or other feature raising high clinical suspicion of TB); medium priority (any of fever <3 weeks, night sweats <4 weeks, measured weight loss <10% in preceding 6 months); low priority = no TB symptoms. ^§^ Screened by research nurse between October 2013 and April 2014. Eligible if not on anti-tuberculosis treatment and persistent or recurrent TB symptoms, defined as: 1) any of cough, fever or night sweats reported at enrolment and any of aforementioned symptoms reported at 3-month visit; OR 2) ⩾5% measured weight loss at 3-month visit and reported unintentional weight loss. XPHACTOR = Xpert for people attending HIV/AIDS care: test or review? HIV = human immunodeficiency virus; ART =antiretroviral therapy; BMI = body mass index; TB = tuberculosis; WHO = World Health Organization.

XPHACTOR study flow, procedures and algorithm are described in detail in the Online Appendix^*^ (section on ‘Main study procedures’, Appendix Figure A.1). Briefly, we enrolled a systematic sample (using a predetermined system designed to minimise the risk of researcher selection bias) of adults (aged ⩾18 years) attending four HIV clinics in Gauteng Province, South Africa, irrespective of the presence of symptoms suggestive of TB. Patients taking anti-tuberculosis treatment within the previous 3 months were excluded. Patients were enrolled into ‘on antiretroviral therapy (ART)’ ‘ART-experienced’ and ‘pre-ART’ (in HIV care or newly diagnosed HIV-positive, not taking ART) groups. At the time of the study, ART eligibility comprised CD4 count ⩽350 cells/mm^3^ or WHO clinical stage ⩾3. Research staff screened participants for TB at monthly intervals up to 3 months using a standardised questionnaire that incorporated the WHO tool. The study algorithm defined individuals as a priori at highest risk of active TB if they had any of the following: current cough, fever ⩾3 weeks, night sweats ⩾4 weeks, body mass index (BMI) <18.5 kg/m^2^, CD4 count <100 cells/mm^3^ or weight loss ⩾10%. A spot sputum sample was collected from these individuals if possible for Xpert testing. At the 3-month visit, all participants underwent sputum (induced if necessary) and blood cultures for mycobacteria (BACTEC MGIT^™^ 960^™^ and 9240^™^ systems; BD, Sparks, MD, USA). We allowed a broad window period around the 3-month XPHACTOR main study visit until around 6 months to maximise follow-up.

### Sub-study eligibility and enrolment

Between October 2013 and April 2014 at the XPHACTOR 3-month visit, consecutive participants who were not on anti-tuberculosis treatment and who had persistent or recurrent symptoms suggestive of TB were invited to participate in this sub-study. Persistent or recurrent TB symptoms were defined as 1) self-report of any of cough, fever or night sweats at enrolment, and self-report of any of the aforementioned symptoms at 3-month visit; or 2) self-report of unintentional weight loss and ⩾5% measured weight loss since XPHACTOR enrolment.

**Figure 1:**
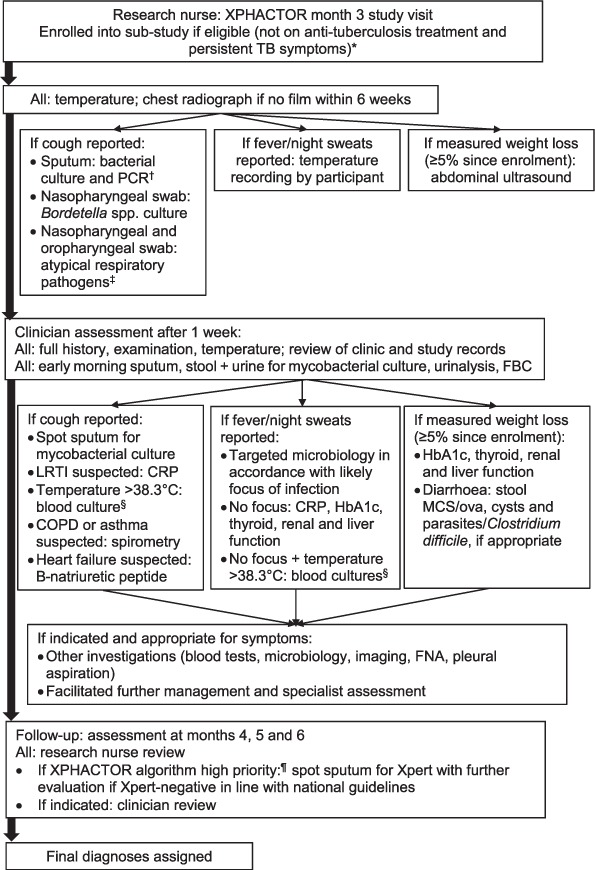
Sub-study procedures. ^*^ Eligible if not on anti-tuberculosis treatment and persistent or recurrent TB symptoms, defined as: 1) any of cough, fever, or night sweats at enrolment and 3-month visit; or 2) ⩾5% measured weight loss at 3-month visit and reported unintentional weight loss. ^†^ Sputum samples underwent macroscopic and microscopic evaluation; culture on 5% horse blood agar (routine respiratory pathogens), Regan Lowe (Bordetella spp.), buffered charcoal yeast extract (Legionella spp.); PCR for Bordetella spp., M. pneumoniae, C. pneumoniae and Legionella spp. ^‡^ PCR for Bordetella spp., M. pneumoniae, C. pneumoniae and Legionella spp. ^§^ Aerobic and anaerobic bacterial cultures. ^¶^ High priority (any of current cough, fever ⩾3 weeks, night sweats ⩾4 weeks, body mass index < 18.5 kg/m^2^, CD4 < 100×10^6^/l, measured weight loss ⩾10% in preceding 6 months, or other feature raising high clinical suspicion of TB); medium priority (any of fever < 3 weeks, night sweats < 4 weeks, measured weight loss < 10% in preceding 6 months); low priority = no TB symptoms. XPHACTOR = Xpert for people attending HIV/AIDS care: test or review? TB = tuberculosis; PCR polymerase chain reaction; FBC = full blood count; LRTI = lower respiratory tract infection; CRP = C-reactive protein; COPD = chronic obstructive pulmonary disease; HbA1c=glycated haemoglobin; MC&S=microscopy, culture and sensitivities; FNA =fine-needle aspiration; HIV = human immunodeficiency virus; AIDS = acquired immune-deficiency syndrome.

Figure 1 shows the sub-study flow and procedures. A chest radiograph (CXR) was requested if there was no film available for the previous 6 weeks, and all were asked to bring samples (stool, early-morning urine and sputum) for mycobacterial culture when they attended for research physician assessment. Further procedures were determined by symptoms (Figure 1); if cough was reported, the research nurse collected an additional sputum sample for bacterial culture (induced if necessary), two nasopharyngeal swabs and one oropharyngeal swab. Sputum samples were tested using routine bacterial microscopy and culture, and polymerase chain reaction (PCR) for bacteria, including Bordetella pertussis. One nasopharyngeal swab was inserted directly into Regan Lowe transport media for Bordetella spp. culture, and the remaining swabs were placed in Primestore medium for PCR detection of B. pertussis and other pathogens (Figure 1). All samples were transported within 24 h of collection to the research laboratory. PCR for B. pertussis was performed in accordance with the method described by Tatti et al.^16^ An abdominal ultrasound scan was requested for those with weight loss. Participants reporting fever or night sweats were given a digital thermometer to record oral temperature (morning, evening, and if any fever or sweats) for 1 week.

### Research physician assessment

**Figure A.2:**
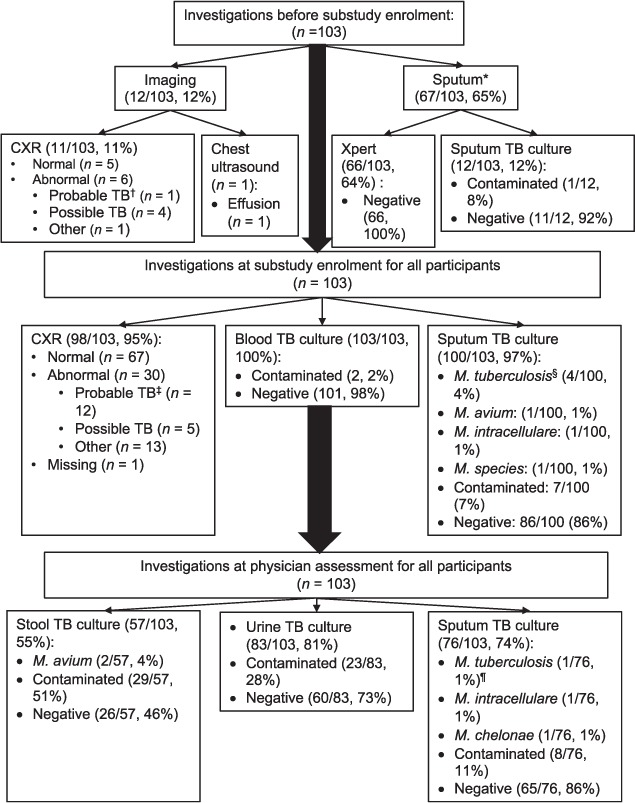
Evaluation for TB undertaken for all study participants. ^*^ At least one sputum for Xpert or mycobacterial culture; 11 participants had both. ^†^ Pleural effusion (n = 1). ^‡^ Pleural effusion (n = 5), upper lobe infiltrates (n = 4), cardiomegaly with ultrasound confirmed pericardial effusion and pleural effusion (n = 1), cardiomegaly with ultrasound confirmed pericardial effusion (n = 1) cavitation and pleural effusion (n = 1). ^§^ Three of the four participants with M. tuberculosis on sputum culture had previously had sputum negative for M. tuberculosis on culture and/or Xpert before sub-study enrolment. ^¶^ Participant had previously had negative sputum for mycobacterial culture at enrolment to sub-study. CXR = chest radiograph; TB = tuberculosis.

Around 1 week after enrolment, sub-study participants underwent systematic clinical evaluation, including examination by a research physician who arranged a standard set of investigations according to the participant's symptoms (Figure 1 and Appendix Figure A.2, Appendix section on ‘Sub-study research physician assessment’).

First-line evaluation for cough was spirometry if cough ⩾8 weeks or features suggestive of chronic obstructive pulmonary disease (COPD) or asthma; if clinically appropriate, blood samples were collected for C-reactive protein (CRP) testing to help distinguish the likelihood of bacterial infection and, if cardiac failure was suspected, for serum β-natriuretic peptide.

Second-line evaluation for cough comprised a trial of appropriate treatment for those with clinical features suggestive of cough due to upper airways disease, angiotensin-converting enzyme (ACE) inhibitors or gastro-oesophageal reflux disease (GORD). All participants were screened using validated tools for depression (Patient Health Questionnaire 9 [PHQ-9]),^17^ household food insecurity (household food insecurity access score [HFIAS]),^18^ and alcohol misuse (Fast Alcohol Screening Test [FAST] score),^19^ and were asked about use of tobacco, snuff and wood-burning stoves. Using a standardised form, the physician abstracted information from clinic records relevant to assigning final diagnoses, such as chronic disease diagnoses, results of recent investigations in particular for TB, and history of HIV, ART and TB.

### Sub-study follow-up

Sub-study participants were followed for a further 3 months and screened for TB at each visit by research staff using a standardised questionnaire incorporating the WHO tool, with further investigation for TB in accordance with the XPHACTOR study algorithm (Figure 1). The research physician reviewed participants at these visits if required to assign final diagnoses.

### Definitions

Final diagnoses were assigned by the research physician using pre-set criteria (Appendix Table A), including the case definitions for TB detailed below.

### TB case definitions

‘Confirmed’ TB was defined as a positive result on 1) Xpert (on sputum sample), 2) line-probe assay (LPA) performed on smear-positive or cultured isolate (GenoType MTBDR*plus*, Hain Lifesciences, Nehren, Germany) or 3) M. tuberculosis culture, from any sample collected within 6 months of sub-study enrolment. ‘Clinical TB’ was defined as anti-tuberculosis treatment started within 6 months of sub-study enrolment in the absence of microbiological confirmation.

### Radiological definitions

‘Probable radiological TB’ was defined as the presence of 1) any of cavitation, predominantly upper lobe infiltrates, pleural or pericardial effusion, or clear miliary picture on CXR, or 2) any of abdominal lymphadenopathy, splenic microabscesses, pleural or pericardial effusion on ultrasound scan. ‘Possible radiological TB’ was defined as the presence of any of lymphadenopathy (hilar or mediastinal), pulmonary nodules or other infiltrates. Participants with ‘probable’ or ‘possible’ radiological TB features but without bacteriological confirmation who started anti-tuberculosis treatment within 6 months of sub-study enrolment were assigned ‘clinical’ TB.

### Ethical approval

The study protocol was approved by the ethics committees of the University of the Witwatersrand, Johannesburg; University of Cape Town, Cape Town, South Africa; and the London School of Hygiene & Tropical Medicine, London, UK. All participants provided written informed consent or, if unable to write, witnessed verbal consent.

## RESULTS

### Sub-study enrolment and eligibility

**Figure 2:**
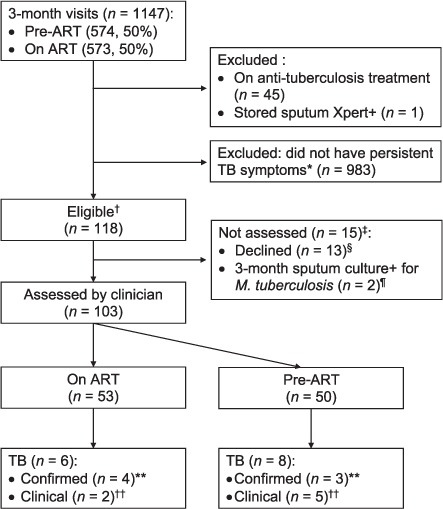
Study flow. ^*^ Persistent TB symptoms, defined as: 1) self-report of any of cough, fever, or night sweats at enrolment and at 3-month visit; or 2) self-report of unintentional weight loss and ⩾5% measured weight loss. ^†^ 60/540 (11%) pre-ART and 58/561 (10%) on ART/eligible. ^‡^ Pre-ART (n = 10); on ART (n = 5). ^§^ No time (n = 12); too unwell (n = 1). ^¶^ Both pre-ART: started anti-tuberculosis treatment before assessment (n = 1); died before assessment (n = 1). ^**^ Confirmed: Xpert+ or LPA+ from smear-positive/cultured isolate OR culture + M. tuberculosis from any sample collected within 6 months from enrolment to sub-study. ^††^Clinical: started treatment in the absence of microbiological confirmation within 6 months from enrolment to sub-study. ART = antiretroviral therapy; += positive; TB = tuberculosis; LPA = line-probe assay.

A total of 1147 XPHACTOR study participants were screened for the sub-study, 45 of whom were excluded because they were currently taking anti-tuberculosis treatment (Figure 2). One further participant was excluded because a stored sputum sample collected for the main study was Xpert-positive when tested after the 3-month visit. Among the remaining 1101 participants, 118 (11%) were eligible, and 103/118 (87%) underwent physician assessment (53/103 [51%] on ART, 50/103 [49%] pre-ART), at a median of 126 days (interquartile range [IQR] 96–175) after enrolment in the parent study. Among 15/118 (13%) participants who did not undergo physician assessment (10 pre-ART, 5 on ART), all had only one symptom (11/15 [73%] ⩾5% measured weight loss, 4/15 [27%] cough), and two subsequently had M. tuberculosis isolated from the 3-month sputum sample.

### Participant characteristics

Table 1 presents the participants' characteristics; 30/50 (60%) pre-ART participants initiated ART during study follow-up, and in the on-ART group, 28/51 (55%) were virologically suppressed. Overall, 40/102 (39%) had PHQ-9 scores suggestive of moderate depression and 53/103 (51%) had HFIAS scores indicating households with severe food insecurity.

**Table 1:** Characteristics of study participants

Characteristic	Pre-ART (*n* = 50) *n* (%)	On ART (*n* = 53) *n* (%)	Total (*n* = 103) *n* (%)
Demographics			
Age, years, median [IQR]	35 [28–41]	45 [39–49]	40 [31–46]
Female	34 (68)	42 (79.3)	76 (73.8)
Black African	50 (100)	49 (92.5)	99 (96.1)
Completed secondary education (grade 12)	15 (30.0)	11 (20.8)	26 (25.2)
Number of people in household, median [IQR]	4 [3–5]	4 [2–5]	4 [2–5]
Monthly household income <2000 ZAR	29 (58.0)	30 (56.6)	59 (57.3)
HIV-TB history			
Duration since HIV diagnosed, months, median [IQR]	6 [4–17]	88 [52–118]	38 [6–99]
ART commenced after enrolment into main study^*^	25 (50.0)	NA	25 (24.3)
Duration on ART, months, median [IQR]	3 [2–4] (*n* = 21)	76 [43–100]	47 [5–88] (*n* = 74)
Previous anti-tuberculosis treatment	4 (8.0)	29 (54.7)	33 (32.0)
>1 previous episode of anti-tuberculosis treatment	0	11 (20.8)	11 (10.7)
CD4^†^/viral load^†^/BMI			
CD4, cells/mm^3^, median [IQR]	311 [162–445] (*n* = 49)	508 [354–673]	401 [226–588] (*n* = 102)
Viral load suppressed (<20 copies/ml)	NA	28 (54.9) (*n* = 51)	29 (53.7) (*n* = 54)
BMI at enrolment to sub-study, kg/m^2^, median [IQR]	22.7 [19.3–26.0]	23.6 [19.6–26.1]	23 [19.4–26.1]
WHO tool symptoms reported			
Unintentional weight loss^‡^ ⩾5%	42 (84.0)	30 (56.6)	72 (69.9)
Cough	15 (30.0)	35 (66.0)	50 (48.5)
Night sweats	10 (20.0)	19 (35.9)	29 (28.2)
Fever	4 (8.0)	18 (34.0)	22 (21.4)
>1 of the above symptoms	17 (34.0)	29 (54.7)	46 (44.7)
Screening for food insecurity and depression			
HFIAS score: severe food insecurity	23 (46.0)	30 (56.6)	53 (51.5)
HFIAS score: moderate food insecurity	11 (22.0)	8 (15.1)	19 (18.5)
PHQ-9 score ⩾10 (moderate depression)	18 (36.0)	22 (42.3) (*n* = 52)	40 (39.2) (*n* = 102)
Tobacco/alcohol/drug use^§^			
Ex- or current smoker	19 (38.0)	26 (49.1)	45 (43.7)
>15 pack years cigarette smoking	3/19 (15.8)	6/26 (23.1)	9/45 (20.0)
Current snuff user	7 (14.0)	15 (28.3)	22 (21.4)
Current harmful alcohol intake (FAST score ⩾3)	9 (18.0)	7 (13.2)	16 (15.5)
Recreational drug use	0 (*n* = 49)	2 (3.8)	2 (2.0)

^*^ A further 5 pre-ART participants initiated ART following clinician assessment.

^†^ Most recent of any result available within 1 year before, or within 6 weeks following clinician assessment.

^‡^ ⩾5% measured weight loss and reported unintentional weight loss at sub-study enrolment.

^§^ ‘Current’ defined as use within past 1 year, and ‘smoker’ defined as having ever smoked ⩾100 cigarettes.

ART = antiretroviral therapy; IQR = interquartile range; ZAR = South African rand; HIV = human immunodeficiency virus; TB = tuberculosis; NA = not applicable; BMI = body mass index; WHO = World Health Organization; HFIAS = Household Food Insecurity Access Score; PHQ-9 = Patient Health Questionnaire 9; FAST = Fast Alcohol Screening Test.

The most common WHO tool symptoms reported were weight loss (83/103, 81%), with 72/103 (70%) having ⩾5% measured weight loss and cough (50/103, 49%); 57/103 (55%) had one WHO tool symptom (45 [44%] weight loss, 9 [9%] cough, 3 [3%] night sweats), and 46/103 (45%) had multiple symptoms (25 [24%] had two, 18 [17%] three, 3 [3%] four symptoms). Among participants reporting cough, 20/50 (40%) had previously received anti-tuberculosis treatment at a median of 4 years (IQR 2–6) before sub-study enrolment (7/50 [14%] more than one course), 29/50 (58%) were current or ex-smokers (7 [14%] had >15 pack years), 8/50 (16%) used snuff, 5/50 (10%) used paraffin stoves and none used wood-burning stoves. A further 18/50 (35%) reported wheeze and 26/50 (52%) dyspnoea.

Among 72 participants with ⩾5% measured weight loss, the median BMI, weight loss and percentage weight loss at physician assessment were respectively 23 kg/m^2^ (IQR 18.9–25.9), 4.4 kg (IQR 3.6–6) and 6.8% (IQR 5.5–9.4). Of these 72 patients, 32 (50%) had HFIAS scores indicating severely food insecure households, 29/71 (41%) had PHQ-9 scores suggestive of moderate depression and 53/72 (74%) had a monthly household income of <2000 South African rand; 67/72 (93%) had follow-up weight measurements, among whom 42/67 (63%) gained weight and in 12/67 (18%) weight was stabilised. Among the 42 participants who gained weight during follow-up, 16/42 (38%) had initiated ART, three of whom had also started anti-tuberculosis treatment. Among 36 participants reporting fever or night sweats, 3/36 (8%) had measured fever >38.3°C at physician assessment or from home measurement.

### Final diagnoses

Table 2 summarises the final diagnoses assigned over a median of 100 days (IQR 89–144) of follow-up. For nine participants (measured weight loss only, *n* = 8; measured weight loss and night sweats, *n* = 1), we were unable to determine any final diagnosis; these patients were assigned a final diagnosis of ‘unexplained’ or ‘unexplained—symptom resolved spontaneously’. One hundred and twenty-one diagnoses were assigned for the remaining 94/103 (91%) participants. The most common diagnoses were weight loss due to severe food insecurity (20/103, 19%), TB (14, 14%), upper respiratory tract infection (12, 12%) and post-TB chronic lung disease (9, 9%).

**Table 2:** Final diagnoses of patients ^*^

Final diagnoses assigned	(*n* = 103) *n* (%)
Weight loss due to severe food insecurity	20 (19.4)
TB	14 (13.6)
Confirmed	7 (6.8)
Clinical	7 (6.8)
Upper respiratory tract infection	12 (11.7)
Post-TB chronic lung disease	9 (8.7)
Bronchiectasis	2 (1.9)
Chronic loculated pleural effusion	1 (1.0)
Likely	6 (5.8)
Weight loss due to loss of appetite	9 (8.7)
Treatment-related^†^	4 (3.9)
Stress-related	4 (3.9)
Unexplained	1 (1.0)
Asthma	7 (6.8)
Confirmed	2 (1.9)
Likely	5 (4.9)
COPD^‡^	5 (4.9)
Confirmed	3 (2.1)
Likely	2 (1.9)
Weight loss due to depression	4 (3.9)
Upper airway cough syndrome	4 (3.9)
Confirmed	3 (2.9)
Likely	1 (1.0)
Perimenopausal vasomotor symptoms	4 (3.9)
Diarrhoeal illness^§^	4 (3.9)
Other infection^¶^	4 (3.9)
Malignancy^#^	3 (2.9)
Alcohol misuse^**^	3 (2.9)
Lower respiratory tract infection	2 (1.9)
Pertussis	2 (1.9)
Confirmed	1 (1.0)
Likely	1 (1.0)
Weight loss due to previously undiagnosed type 2 diabetes	2 (1.9)
Weight loss due to end-stage renal disease	2 (1.9)
Other^††^	11 (10.7)
Unexplained	10 (9.7)
Unexplained - symptom resolved spontaneously	9 (8.7)

^*^ 140 diagnoses were assigned for 103 participants: 70 (68%) had one final diagnosis, 29 (28%) two diagnoses and 4 (4%) had three diagnoses.

^†^ Attributed to new ART regimen (*n* = 2), radiotherapy for Kaposi's sarcoma (*n* = 1), dental extraction (*n* = 1).

^‡^ One participant with likely COPD had a previous addiction to nyaope (a street drug that is smoked and reported to contain heroin, cannabis and antiretrovirals); one participant with confirmed COPD had clinical cor pulmonale.

^§^ Microbiological confirmation of isospora (*n* = 1), giardia (*n* = 1); cause not known (*n* = 2).

^¶^ Escherichia coli urinary tract infection (*n* = 2), chronic skin infection (*n* = 1), likely chronic pelvic infection (*n* = 1).

^#^ Newly diagnosed Hodgkin's lymphoma (*n* = 1), progression of previously diagnosed malignancy (renal cell carcinoma, *n* = 1; cervical cancer, *n* = 1).

^**^ Hazardous alcohol intake and weight loss after stopping (*n* = 1), no other cause identified for night sweats (*n* = 2).

^††^ Diagnoses for cough, *n* = 5 (ACE inhibitor-related, *n* = 1; GORD-related, *n* = 1; post-thoracic surgery for benign lung mass, *n* = 1; post-infectious, *n* = 1; smoking-related, *n* = 1). Diagnoses for weight loss, *n* = 6 (endoscopy-confirmed gastritis, *n* = 1; recurrent small bowel obstruction, *n* = 1; confirmed heart failure, *n* = 1; subclinical hyperthyroidism, *n* = 1; increased exercise, *n* = 1; chronic unexplained gastrointestinal symptoms resolved by end of study, *n* = 1).

TB = tuberculosis; COPD = chronic obstructive pulmonary disease; ART = antiretroviral therapy; ACE = angiotensin-converting enzyme; GORD = gastroesophageal reflux disease.

Table 3 summarises the final diagnoses for the most common symptoms reported: cough and ⩾5% measured weight loss. Among 50 participants reporting cough, the most common diagnoses were upper or lower respiratory tract infection (11/50, 22%), post-tuberculous chronic lung disease (9/50, 18%), TB (7/50, 14%: pulmonary only, *n* = 4; extra-pulmonary only, *n* = 1; both, *n* = 2), asthma (7/50, 14%), COPD (5/50, 10%) and upper airways cough syndrome (4/50, 8%). Samples collected from 40 participants for respiratory pathogens yielded only one positive sample that was PCR-positive for B. pertussis.

**Table 3:** Final diagnoses of participants with cough and/or weight loss (n = 100)

	Cough only (*n* = 28)	Cough and weight loss (*n* = 22)^*^	Weight loss only (*n* = 50)
Severe food insecurity	—	6	14
Asthma or COPD	10	2	—
Unexplained weight loss	—	2	9
Upper or lower respiratory tract infection	5	6	—
TB	2	5	5
Pulmonary	2	2	2
Extra-pulmonary	—	1	2
Both	—	2	1
Post-TB chronic lung disease	4	5	—
Weight loss due to loss of appetite^†^	—	3	5
Weight loss due to depression	—	2	1
Diarrhoeal illness	—	—	3
Malignancy^‡^	—	—	3
Upper airway cough syndrome	2	2	—
Pertussis^§^	2	—	—
Weight loss due to previously undiagnosed type 2 diabetes	—	1	1
End-stage renal disease	—	—	2
Other^¶^	3	4	7

^*^ 38 final diagnoses made for 22 participants with both cough and weight loss

^†^ Weight loss only, *n* = 3: stress-related, *n* = 2; unexplained, *n* = 1; both cough and weight loss, *n* = 5: treatment-related, *n* = 3; stress-related, *n* = 2.

^‡^ Newly diagnosed Hodgkin's lymphoma, *n*=1; progression in previously diagnosed malignancy (renal cell carcinoma, *n*=1; cervical cancer, *n*= 1).

^§^ One confirmed, and one likely.

^¶^ 1) Cough only, *n* = 3: GORD-related, *n* = 1; post-thoracic surgery for benign lung mass, *n* = 1; smoking-related, *n* = 1. 2). Weight loss only, *n* = 7: endoscopy-confirmed gastritis, *n* = 1; confirmed heart failure, *n* = 1; subclinical hyperthyroidism, *n* = 1; increased exercise, *n* = 1; chronic unexplained gastrointestinal symptoms resolving by end of study, *n* = 1; likely chronic pelvic infection, *n* = 1; hazardous alcohol intake and weight loss after stopping, *n* = 1. 3). Both cough and weight loss, *n* = 4: ACE inhibitor-related cough, *n* = 1; post-infectious cough, *n* = 1; weight loss likely due to recurrent small bowel obstruction, *n* = 1; weight loss due to unexplained loss of appetite, *n* = 1.

COPD = chronic obstructive pulmonary disease; TB = tuberculosis; GORD = gastro-oesophageal reflux disease; ACE = angiotensin-converting enzyme.

Among the 72 participants with measured weight loss, the most common diagnoses were weight loss due to severe food insecurity (20/72, 28%), TB (10/72, 14%: pulmonary only, *n* = 3; extra-pulmonary only, *n* =4; both, *n*=3) and prolonged loss of appetite (8/72, 11%: 4 due to medical treatment and 4 stress-related). The most common diagnosis among 45 participants with weight loss in the absence of other symptoms was severe food insecurity (13/45, 29%). Of the 72 patients, 11 (15%) had weight loss unexplained by study investigations, among whom four gained weight after ART initiation. Nine of 68 participants (13%) with available samples had glycated haemoglobin ⩾6.5%; in two of these weight loss was attributed to newly diagnosed type 2 diabetes mellitus. Thyroid function test abnormalities were newly identified in 18/67 (27%); one participant with biochemically subclinical hyperthyroidism (high thyroxine, normal thyroid stimulating hormone) and marked weight loss (18 kg) also had severe food insecurity. Abdominal ultrasound scans were abnormal for 22/65 (34%), five of whom had features compatible with probable radiological TB and 17 other abnormalities (gallstones, *n* = 5; hepatomegaly, *n* = 4; echogenic kidney or liver, *n* = 3; splenomegaly, *n* = 2; metastatic disease, *n* = 2; fatty liver, *n* = 1).

### Tuberculosis diagnoses

Appendix Figure A.2 gives the results of the mycobacteriology and radiology requested for all sub-study participants. All participants had at least one sample subjected to mycobacterial culture; culture was positive for M. tuberculosis in 5/103 (5%) participants (5/176, 3% sputum samples [one multidrug-resistant]; 0/103 blood, 0/83 urine and 0/57 stool). Of 98 CXRs, 17 (17%) fulfilled the criteria for radiological TB (probable radiological TB, *n* = 12; possible radiological TB, *n* = 5); 6/17 (35%) participants whose CXRs fulfilled the criteria for radiological TB also fulfilled TB case definitions (3 confirmed, 3 clinical). Of 65 abdominal ultrasound scans, 7 (11%) fulfilled the criteria for probable radiological TB (abdominal only, *n* = 4; abdominal and possible renal, *n* = 1; pericardial effusion and abdominal, *n* = 1; pleural and pericardial effusions and abdominal TB, *n* = 1); 6/7 (86%) participants whose abdominal ultrasound scans fulfilled the criteria for radiological TB also fulfilled TB case definitions (3 confirmed, 3 clinical).

Of 103 sub-study participants, 14 (14%) (6 on ART, 8 pre-ART) fulfilled TB case definitions (7 confirmed, 7 clinical). Eight participants started treatment due to compatible imaging (4 ultrasound, 2 abdominal ultrasound and CXR, and 2 CXR), of whom 3 were subsequently bacteriologically confirmed on sputum (1 Xpert + culture, 2 culture). Four participants started anti-tuberculosis treatment based on a positive sputum result (2 Xpert, 2 culture). One participant started treatment based on histology following fine-needle lymph node aspiration, and one based on lumbar puncture.

The median time from enrolment to start of anti-tuberculosis treatment was 21 days (range 1–137) for 13 participants with a documented anti-tuberculosis treatment start date. One further participant had positive Xpert on sputum 149 days after enrolment but an unknown treatment start date. Among the 8 pre-ART participants, 4 started anti-tuberculosis treatment after ART initiation (3 within 3 months, 1 within 6 months). A further two participants who were enrolled but who did not undergo physician assessment fulfilled case definitions for confirmed TB, of whom one died before anti-tuberculosis treatment was initiated (Figure 2).

## DISCUSSION

In this representative sample of HIV clinic attendees in South Africa reporting persistent or recurrent WHO tool symptoms 3 months after a negative initial investigation for TB, among those able to produce sputum, 14/103 (14%) had TB. Half started anti-tuberculosis treatment based on imaging, mainly abdominal ultrasound, which illustrated the limitations of sputum-based diagnostics for detecting extra-pulmonary TB. With an estimated 40% shortfall globally between notified cases and estimated incidence of TB in 2016, and with South Africa one of the 10 countries accounting for most of this gap,^20^ we recommend using multiple diagnostic modalities, particularly imaging, to help identify these missing TB patients.

Our study is the first to systemically evaluate patients established in HIV care with persistent or recurrent symptoms suggestive of TB, and with an initial negative Xpert result among those able to produce sputum, for a broad spectrum of diagnoses. Previous studies have investigated patients with persistent symptom(s) for specific infectious^5^,^7^ or non-communicable causes,^6^,^8^,^14^ or evaluated chronic cough in smear-negative patients before the roll-out of Xpert.^9^,^12^ Munyati et al. evaluated primary care attendees in Zimbabwe with chronic cough and, unsurprisingly, among 454 newly diagnosed HIV-positive patients, the majority of the diagnoses were infectious (TB, 46%; lower respiratory tract infection, 31%).^21^ Munyati et al. also identified a high proportion of non-communicable disease diagnoses, in particular post-tuberculous disease, asthma and heart failure.^21^ We also found post-TB chronic lung disease to be a relatively common diagnosis; better criteria to distinguish it from active TB and optimal management are needed.^22–24^ Our data support Chakaya et al.'s call for large-scale epidemiological studies of post-TB lung disease.^22^

Severe food insecurity was the most common cause of weight loss. Food insecurity has not previously been evaluated as a possible cause for weight loss in the context of TB screening, although it is well described as a barrier to adherence to ART.^25^ We only assigned this diagnosis after searching for other, more likely diagnoses, and chose severe (rather than moderate) food insecurity as a more specific marker. Clinicians should consider screening for food insecurity among people with weight loss, particularly if not associated with other symptoms, and ensure patients are linked to social support where available. Forty per cent of our study participants screened positive for significant depression, and almost one fifth had harmful alcohol use, comparable with estimates of 31% and 7–31%, respectively, from a systematic review in sub-Saharan Africa of HIV-positive people on ART by Nakimuli-Mpungu et al.^26^ In their pooled analysis, individuals with significant depression were less likely to adhere to ART.^26^ Screening for depression with provision of appropriate care should be part of the HIV care package in lower-income settings to help optimise ART adherence and treatment outcomes.

The WHO tool was developed for use in resource-limited settings to provide a simple clinical algorithm to reliably rule out TB before providing isoniazid preventive therapy to PLHIV. As the tool was designed to maximise sensitivity (78.9%) and minimise the negative likelihood ratio for TB, it has low specificity (49.6%). At a TB prevalence of 5% in PLHIV, it has a negative predictive value of 97.7%, but a very low positive predictive value (8%).^27^ Individuals who screen positive, the majority of whom will not have TB, require further evaluation for TB using Xpert, which has been recommended as the initial diagnostic test.^1^ This poses a huge challenge in resource-constrained settings when it is used, as recommended, for active TB case finding in PLHIV at every clinical encounter.^1^ Simple, low-cost strategies to prioritise those with WHO tool symptoms for TB investigation, such as ‘second-step’ clinical algorithms,^15^,^28^ or point-of-care CRP testing (also suggested as an alternative TB screening tool),^29^ are potential solutions that merit further evaluation.

Strengths of our study included our systematic physician evaluation of a representative sample of HIV clinic attendees in a clinically relevant manner with a standardised set of investigations and longitudinal follow-up of participants. We cannot rule out that additional diagnosis of TB and other specific diagnoses might have been made if further investigations had been undertaken. Weight loss was commonly reported by our study participants, but we restricted our study to those with measured weight loss to make this criterion more objective.

## CONCLUSIONS

TB, post-TB chronic lung disease and food insecurity were the main diagnoses for symptoms suggestive of TB in our population of HIV clinic attendees who had previously undergone systematic screening and investigation for TB, and we were able to assign diagnoses for more than 90% of participants. Our study highlights the need to continue to investigate for TB using multiple modalities among HIV-positive people with persistent symptoms, as well as evaluation for food insecurity, and for further studies to guide the identification and management of the sequelae of pulmonary TB.

## Footnotes

^*^ The appendix is available in the online version of this article, at http://www.ingentaconnect.com/content/iuatld/ijtld/2019/00000023/00000002/art000.....

## APPENDIX

### MAIN STUDY PROCEDURES FOR XPHACTOR

#### Enrolment

At enrolment, research staff administered a standardised questionnaire, which incorporated the World Health Organization (WHO) tool, collected details of tuberculosis (TB) and human immunodeficiency virus (HIV) treatment, and basic demographic and socioeconomic information. Further investigation was prioritised according to the XPHACTOR (‘Xpert for people attending HIV/AIDS care: test or review?’) algorithm with an immediate spot sputum sample sent for Xpert^®^ MTB/RIF (Cepheid, Sunnyvale, CA, USA) testing for individuals at a priori highest risk of active TB: 1) all assigned ‘high priority’ (any of the following: current cough, fever ⩾3 weeks, body mass index [BMI] <18.5 kg/m^2^, CD4 count < 100×10^6^/l, measured weight loss ⩾10% in preceding 6 months or other feature raising high clinical suspicion of TB); 2) those in the pre-ART group with CD4 count of <200×10^6^/l at enrolment; 3) all those in the HIV testing and counselling (HTC) group (whose CD4 count was unknown) at enrolment. For all other participants, a spot sputum sample was collected at enrolment and frozen at −80°C within 24 h for testing with Xpert at the end of the study (Figure A.1). This enabled comparison of sensitivity and specificity of the XPHACTOR study algorithm to detect TB cases against sensitivity and specificity if Xpert had been performed immediately for all with any WHO tool symptom.

#### Follow-up

Participants were reviewed monthly to 3 months, with repeat WHO symptom screen and a spot sputum requested for Xpert if ‘high priority’ by the study algorithm at that visit, with the exception of those in the ‘on ART’ group who were asymptomatic at enrolment, who were telephoned at 1 and 2 months to update locator information but were not asked about TB symptoms. At the 3-month visit, sputum (induced if necessary) and blood were collected for mycobacterial culture on liquid media (BACTEC MGIT^™^ [Mycobacterium Growth Indicator Tubes; BD, Sparks, MD, USA] 960^™^ and 9240^™^ systems) from all study participants, regardless of symptoms (Figure A.1). We allowed a broad window period around the scheduled 3-month visit until around 6 months to maximise study follow-up.

Participants who submitted an Xpert sample were reviewed within 1 week. If Xpert-positive, anti-tuberculosis treatment was initiated; if negative, research staff repeated WHO symptom screening and facilitated the Xpert-negative algorithm, which comprised chest radiograph (CXR), spot sputum for TB culture and/or antibiotic trial as clinically appropriate (Figure A.1).

Investigation results were returned to clinic staff, who were responsible for management decisions. Clinic records were reviewed at the end of the study to ascertain any additional relevant investigations and/or TB diagnoses. Deaths were identified through reports from participant-nominated contacts, clinic staff, and by accessing the Department of Home Affairs vital statistics database using participants' South African identification (ID) numbers.

#### Sub-study research physician assessment

The research physician administered a standardised questionnaire which had targeted questions to systematically identify the cause for WHO tool symptom(s) reported. All participants were asked about past medical history, current medications, and investigations and treatment undertaken to date. For example, those with cough were asked about cough duration and triggers, associated symptoms and any preceding respiratory tract infection. Results for all previous investigations undertaken for the study were reviewed by the research physician, for example, CXR and abdominal ultrasound scan; Xpert and mycobacterial culture on sputum; and sputum, nasopharyngeal and oropharyngeal samples for respiratory pathogens. Participants with pleural effusions or lymphadenopathy were referred to the clinic physician for consideration for aspiration or fine-needle aspiration, as appropriate. Symptom-specific evaluation undertaken by the research physician is summarised below.

### EVALUATION OF COUGH

#### First-line evaluation

Blood samples were collected from all participants with cough for full blood count and, where appropriate, C-reactive protein (CRP), to help distinguish the likelihood of bacterial respiratory infection and, if febrile (>38.3°C), aerobic and anaerobic bacterial blood cultures. If bacterial infection was suspected, oral antibiotics or hospital admission were facilitated when clinically appropriate.

A trained research physician performed spirometry in accordance with American Thoracic Society (ATS) and European Respiratory Society (ERS) standards^1^ for any participant with cough ⩾8 weeks, or features suggestive of chronic obstructive pulmonary disease (COPD) or asthma, unless respiratory clinic spirometry results were already available. The Advanced Medical Engineering spirometer (AME, Cape Town, South Africa) was used, with calibration checks performed in accordance with the manufacturer's recommendations. Up to eight seated readings were taken, and post-bronchodilator (5 mg nebulised salbutamol) spirometry performed if a spirometry abnormality was found. If ATS/ERS within- and between-manoeuvre acceptability criteria were not met, we reported usable curves (good start and satisfactory exhalation). Post-bronchodilator spirometry data were used to confirm airflow obstruction, defined using Global Lung Initiative (GLI) 2012 equations (forced expiratory volume in 1 s [FEV_1_]/forced vital capacity [FVC] < lower limit of normal at the 5^th^ centile).^2^ Post-bronchodilator increase in FEV_1_ >12% of predicted and >200 ml was used to confirm asthma. Participants were referred to the clinic physician for further management if spirometry confirmed asthma or COPD, or if spirometry was normal but obstructive airways disease likely, or if another spirometry abnormality was identified. Response to any treatment provided was assessed at 4–12 weeks.

If participants had clinical features suggestive of cardiac failure, serum β-natriuretic peptide (BNP) was measured and if levels were >100 pg/ml, further cardiological evaluation and management was facilitated. If participants had symptoms compatible with Pneumocystis jiroveci pneumonia (fever/exertional dyspnoea/tachypnoea) and CD4 count <200 cells/μl, exercise oximetry was undertaken and the participant referred to the responsible clinic physician for further management.

Any suspicious CXR features were discussed with the clinic physician to facilitate further appropriate evaluation or treatment as deemed appropriate, for example, pleural aspiration, computed tomography (CT) imaging, endoscopy or bronchoscopy or presumptive anti-tuberculosis treatment.

#### Second-line evaluation of cough

If diagnosis for cough was not identified by first-line evaluation, a trial of appropriate treatment was arranged for those with clinical features suggestive of cough due to upper airways disease, angiotensin-converting enzyme (ACE) inhibitors or gastrooesophageal reflux (GORD). This comprised corticosteroid nasal spray and/or antihistamine, or ear, nose, throat referral if upper airways disease was suspected; switching ACE inhibitor to a suitable alternative; lifestyle advice and trial of a proton pump inhibitor if GORD was suspected. Response to treatment was reviewed at 4–12 weeks. Smoking cessation advice was given to all current smokers, and improvement in cough evaluated at 4–12 weeks post-cessation, if applicable. If no likely cause for cough was identified after second-line evaluation, referral to a respiratory physician was facilitated.

#### Evaluation of ⩾5% unintentional weight loss

Evaluation aimed to identify a broad spectrum of causes of weight loss, including TB, endocrine disorders, malignancy, depression, inadequate access to food and drug misuse.

**Table A:** Criteria for diagnoses assigned for WHO tool symptoms

Diagnosis	Criteria
Cough	
Asthma	Likely: compatible symptoms (recurrent or chronic cough,^*^ wheeze, dyspnoea, chest tightness) without spirometry confirmation;
	Confirmed: compatible symptoms confirmed using spirometry (GLI criteria,^†^ and improvement in FEV_1_ after bronchodilator, >12% predicted and >200 ml); OR documented diagnosis
COPD	Age >35 years and risk factor (smoking or snuff use or exposure to biomass fuel); AND Likely: compatible symptoms (chronic cough,^*^ wheeze, dyspnoea, sputum production, frequent bronchitis) and either borderline FEV_1_/FVC on spirometry or absence of spirometry confirmation
	Confirmed: compatible symptoms confirmed using spirometry;^†^ OR documented diagnosis
	Not COPD: compatible features but normal spirometry
Post-TB CLD	Post-TB bronchiectasis: chronic^*^ productive cough with compatible chest CT scan; OR documented diagnosis
	Chronic loculated pleural effusion post-TB: documented diagnosis with compatible chest ultrasound or CT scan
	Likely post-TB CLD: chronic cough^*^ or recurrent respiratory tract infections^‡^ in patient previously treated for TB, and/or CXR abnormality compatible with previous pulmonary TB (fibrosis, hyperinflation, bronchovascular distortion, bronchiectasis)
Lower respiratory tract infection	Symptoms for ⩽3 week duration, and:
	Cough and ⩾1 lower respiratory tract symptom (fever, sputum production, breathlessness, wheeze, chest discomfort or pain)
Upper respiratory tract infection	Symptoms for ⩽3 weeks' duration, and includes:
	Cold: ⩾1 of cough, nasal symptoms, sneezing, sore throat;
	Influenza: fever and ⩾1 of headache, myalgia, cough or sore throat
Pertussis	Acute cough ⩾2 weeks and symptomatic (⩾1 of: paroxysms of cough, post-tussive vomiting or inspiratory whoop)
	Likely: no microbiological confirmation and no other likely cause
	Confirmed: respiratory sample positive for Bordetella pertussis
Post-infectious cough	Cough duration 3–8 weeks preceded by acute respiratory tract infection and no other likely cause
Gastro-oesophageal reflux disease	Compatible symptoms (chronic cough worse with or after meals associated with heartburn or regurgitation, dysphonia, cough resolves during sleep) improving within 3 months with appropriate treatment
Upper airway cough syndrome	Chronic^*^ or recurrent cough and compatible symptoms (post-nasal drip, nasal discharge or congestion, catarrh) responding to appropriate treatment; OR documented diagnosis
	Likely: symptoms as above but no trial of treatment, and no other likely cause
ACE inhibitor cough	Chronic^*^ dry cough resolving within 12 weeks of stopping ACE
Tobacco-related cough	Current smoker or snuff user and no other likely cause for cough
Unintentional weight loss	
Malignancy	Compatible clinical features with confirmatory imaging or histology; OR documented diagnosis
Food insecurity	Compatible HFIAS score (severe food insecurity)
Type 2 diabetes	HbA1c ⩾48 mmol/mol (6.5%) and compatible symptoms
Hyperthyroidism	Low TSH with raised free thyroxine level
	Subclinical/biochemical: low TSH with normal free thyroxine level
Depression	Compatible history and PHQ-9 score ⩾10; OR documented diagnosis
Stress-related	History of loss of appetite triggered by acute stressful event
Treatment-related	History of loss of appetite/reduced food intake due to side effects of medical treatment (e.g., switch to second-line ART)
Fever/night sweats	
Infection	Likely: compatible symptoms responding to appropriate treatment or resolving spontaneously
	Confirmed: compatible symptoms with microbiological confirmation
Perimenopause	Compatible symptoms, confirmed with FSH if age <45 years, and no other likely cause
Diagnoses relevant to >1 WHO tool symptom	
Confirmed TB	Positive result on 1) Xpert or 2) line-probe assay or 3) M. tuberculosis culture, from any sample collected within 6 months of sub-study enrolment
Clinical TB	Anti-tuberculosis treatment started within 6 months of sub-study enrolment in the absence of microbiological confirmation
Heart failure^§^	Likely: compatible symptoms (orthopnoea, dyspnoea, peripheral oedema) and serum BNP > 100 pg/ml
	Confirmed: compatible symptoms and echocardiogram; OR documented diagnosis
Alcohol misuse^¶^	Hazardous alcohol consumption (FAST score ⩾3) and no other likely cause
Unexplained, symptom resolved spontaneously	No cause for symptom identified, and symptoms resolved by final study visit
Unexplained	No cause identified

^*^ Defined as cough ⩾8 weeks.

^†^ Defined as no improvement in FEV_1_ after bronchodilator and either 1) GLI^2^ equation criterion FEV_1_/FVC <lower limit of normal, or 2) GOLD FEV_1_/FVC < 0.7. GOLD criteria were used in spirometry results from medical records.

^‡^ Defined as ⩾2 episodes in preceding 6 months.

^§^ Considered as possible cause of chronic cough or weight loss.

^¶^ Considered as possible cause of weight loss or sweats on withdrawal.

WHO = World Health Organization; GLI = Global Lung Initiative; FEV_1_ = forced expiratory volume in 1 s; COPD = Chronic Obstructive Lung Disease; FVC = forced vital capacity; TB = tuberculosis; CLD = chronic lung disease; CT =computed tomography; CXR = chest radiograph; ACE = angiotensin-converting enzyme; HFIAS = Household Food Insecurity Access Score; HbA1c = glycated haemoglobin; TSH = thyroid stimulating hormone; PHQ9 = Patient Health Questionnaire 9; ART = antiretroviral therapy; FSH = follicle stimulating hormone; BNP = brain natriuretic peptide; FAST = Fast Alcohol Screening Test; GOLD = Global Initiative for Chronic Obstructive Lung Disease.

Blood samples were collected from all participants with weight loss for renal, liver and thyroid function, full blood count and glycated haemoglobin (HbA1c; to identify type 2 diabetes mellitus), and clinically significant results were reported to the clinic physician for further management. If participants reported diarrhoea, stool samples were collected for microscopy, bacterial culture, parasitology and for Clostridium difficile, if antibiotics had been taken in the preceding 12 weeks.

The Patient Health Questionnaire-9 (PHQ-9)^3^ was used to screen all sub-study participants for depression. This score categorises depression as 0–4 (none or minimal), 5–9 (mild), 10–14 (moderate), 15–19 (moderately severe) and 20–27 (severe). Participants with scores of ⩾10 were evaluated further by the research physician, and referred to the clinic physician or psychology service if deemed clinically depressed. The Fast Alcohol Screening Test (FAST)^4^ was used to screen for hazardous alcohol consumption (FAST score ⩾3) and all participants were asked about drug misuse. If FAST score was ⩾3, brief intervention was provided and, if appropriate, participants were referred to drug services.

We used the Household Food Insecurity Access Scale (HFIAS)^5^ to measure food access, categorising the scale as 1) ‘food secure’, 2) ‘mildly food insecure access’, 3) ‘moderately food insecure access’ or 4) ‘severely food insecure access’. A HFIAS score of 4 was deemed a cause of unintentional weight loss. Participants with food insecurity were referred to the clinic dietician.

Clinical features suggestive of possible malignancy were discussed with the clinic physician to facilitate further appropriate evaluation or treatment as deemed appropriate.

#### Evaluation of fever or night sweats

Self-recorded participant temperature measurements were reviewed. If a likely focus of infection was identified, then relevant samples were submitted for appropriate microbiological evaluation such as midstream urine (bacterial culture), stool (bacterial culture, parasitology, Clostridium difficile), sputum (bacterial culture), blood (malaria film) or swabs (bacterial culture). If deemed clinically appropriate, antibiotics were provided and the participant reviewed to assess response to treatment.

If no likely focus infection was identified, blood was collected for renal, liver and thyroid function, full blood count, CRP, HbA1c, aerobic and anaerobic culture if documented fever >38.3°C; and urine for microscopy and culture. Abdominal ultrasound was arranged and CXR, if either no recent film or no film since onset of symptoms.

A clinical diagnosis was made if no other more likely cause was identified, and symptoms were suggestive of perimenopausal vasomotor symptoms in females aged ⩾45 years; in younger cases, blood samples were collected to determine follicle-stimulating hormone (FSH) levels.

If, during in-patient treatment, specialist referral or further evaluation such as lumbar puncture, CT imaging (abdomen, chest, sinus), fine-needle aspiration, bone-marrow aspiration or pleural aspiration were deemed necessary, this was facilitated by the clinic physician.
